# Artificial light pollution: are shifting spectral signatures changing the balance of species interactions?

**DOI:** 10.1111/gcb.12166

**Published:** 2013-03-12

**Authors:** Thomas W Davies, Jonathan Bennie, Richard Inger, Natalie Hempel Ibarra, Kevin J Gaston

**Affiliations:** * Environment and Sustainability Institute, University of ExeterPenryn, Cornwall, TR10 9EZ, UK; † Centre for Research in Animal Behaviour, School of Psychology, University of ExeterExeter, Devon, EX4 4QG, UK

**Keywords:** animals, artificial light spectra, pollution, species interactions, street lighting, vision ecology

## Abstract

Technological developments in municipal lighting are altering the spectral characteristics of artificially lit habitats. Little is yet known of the biological consequences of such changes, although a variety of animal behaviours are dependent on detecting the spectral signature of light reflected from objects. Using previously published wavelengths of peak visual pigment absorbance, we compared how four alternative street lamp technologies affect the visual abilities of 213 species of arachnid, insect, bird, reptile and mammal by producing different wavelength ranges of light to which they are visually sensitive. The proportion of the visually detectable region of the light spectrum emitted by each lamp was compared to provide an indication of how different technologies are likely to facilitate visually guided behaviours such as detecting objects in the environment. Compared to narrow spectrum lamps, broad spectrum technologies enable animals to detect objects that reflect light over more of the spectrum to which they are sensitive and, importantly, create greater disparities in this ability between major taxonomic groups. The introduction of broad spectrum street lamps could therefore alter the balance of species interactions in the artificially lit environment.

## Introduction

Artificial lights have been used to illuminate the night-time environment for over a century, during which numerous alternative lighting technologies have arisen, each emitting light with unique spectral characteristics (Elvidge *et al*., 2010). Now widely distributed, artificial lighting is spreading at a rate of 6% per year globally (Hölker *et al*., 2010). Indeed, by 2001 it was estimated that the fraction of land under skies that were artificially brightened above natural background levels already exceeded 10% in 66 nations across the planet (Cinzano *et al*., 2001). Organisms have evolved under the intensities, timings and spectral composition of light emitted from the sun and stars, and reflected from the moon. However, artificial lighting is changing all these aspects of natural light regimes (Gaston *et al*., 2012) leading to a potentially diverse array of ecological impacts (Longcore & Rich, 2004; Perkin *et al*., 2011; Gaston *et al*., 2013). A recent surge in research activity has revealed that artificially lighting the nocturnal environment can have impacts ranging from changes in animal behaviour (Rydell, 1992; Bird *et al*., 2004; Eisenbeis, 2006; Stone *et al*., 2009; Titulaer *et al*., 2012) to the composition of whole communities (Davies *et al*., 2012). Yet, because artificial light pollution has only recently become widely recognized as an environmental issue, studies on its ecological effects remain relatively scarce.

Since the second half of the 20th century, narrow spectrum Low Pressure Sodium (LPS) lighting, with a characteristic orange hue, has been the most common form of street lighting in many regions. However, ongoing advances in street lighting technology have led to the increasing adoption of broader spectrum light sources such as High Pressure Sodium (HPS), Light Emitting Diode (LED) and Metal Halide (MH) lamps (Elvidge *et al*., 2010), which provide improved colour rendering capabilities for humans. Shifting and broadening the spectra of street lamps may lead to unforeseen environmental impacts because the spectral signature reflected from objects is an important cue that guides a number of animal behaviours, including, for example, the detection of resources (Chittka *et al*., 1994; Hempel de Ibarra & Vorobyev, 2009; Macedonia *et al*., 2009; Chiao *et al*., 2011; Zou *et al*., 2011), mate selection (Andersson *et al*., 1998; Hunt *et al*., 2001; Robertson & Monteiro, 2005; Lim *et al*., 2008) and navigation (Cheng *et al*., 1986; Möller, 2002; Mappes & Homberg, 2004; Reppert *et al*., 2004; Ugolini *et al*., 2005). Here, we ask whether the use of broad spectrum street lighting technologies is likely to improve the ability of animals to perform tasks during the night which are guided by the detection of light reflected from objects, and whether this could alter the balance of species interactions.

## Materials and methods

### Overview

**Fig. 1:**
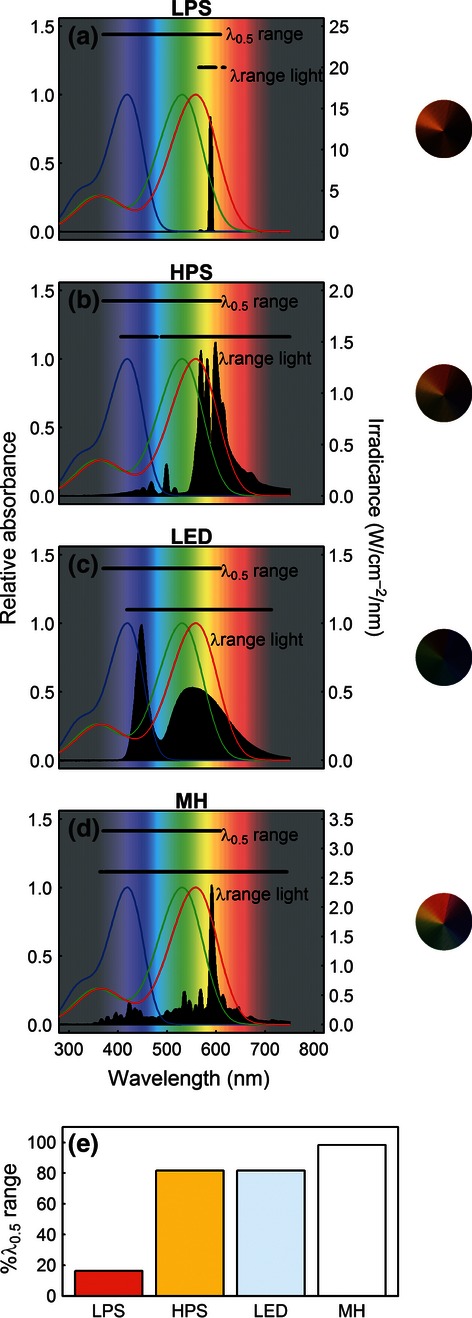
The colour vision performance of human beings under light emitted from four contrasting street lighting technologies. (a). LPS lamps which emit light over a narrow region of the light spectrum (λ_range_ light) stimulate a smaller proportion of the region of the light spectrum to which human visual pigments are half maximally sensitive (λ_0.5_ range) (dashed line), hence objects which reflect light outside of this range appear less bright (colour wheel insert). (b,c,d). Broad spectrum street lighting technologies (HPS, LED, MH) emit light across a broader region of the light spectrum to which humans are sensitive, allowing us to identify objects reflecting light across a broader range of wavelengths. (e). The visual performance of humans under each of the street lighting types can be compared using an index (% λ_0.5_ range stimulated) calculated as the percentage of λ_0.5_ range overlapped by λ_range_ light. A–D. Solid black lines represent the α and β band absorbance curves for the three visual pigments used to detect light in the human visual system. The emission spectrum of each street light is represented by the filled curve. The plot background approximates the colour of the light detected at each wavelength by the human visual system. UV light is emitted below 400 nm and infrared light above 700 nm. Colour wheel inserts are photographic images taken of the same colour wheel under each of the street lighting types using a standard digital SLR camera which detects red, green and blue light at approximately the same wavelengths as human visual pigments are maximally sensitive.

We based our analysis on a novel collation of previously published wavelengths at which the visual pigments contained within the photoreceptors of 213 species of animal (comprising 7 arachnids, 112 insects, 16 birds, 32 reptiles and 46 mammals) maximally absorb light (λ_max_) (see Table S1). Using a previously derived formula which describes the absorbance properties of visual pigments based on their λ_max_ (see Govardovskii *et al*., 2000 for details), we then modelled the absorbance of the visual pigments in each species from 200 to 750 nm and estimated the maximum (maxλ_0.5_) and minimum (minλ_0.5_) wavelengths of half maximum absorbance (Fig. 1) to determine the range of wavelengths detectable by each species (λ_0.5_ range). By comparing the region of the light spectrum over which LPS, HPS, LED and MH lamps emit light (λ_light_ range) with the region of the light spectrum over which the visual pigments in animal eyes absorb more than half of the light passing through them (λ_0.5_ range), we obtained a value of the proportion of the visually detectable wavelength range at greater than half maximum absorbance which is stimulated by each type of street lamp (% λ_0.5_ range). By way of example, Fig. 1 illustrates how the % λ_0.5_ range of the visual system in humans relates to the objects we can detect in the environment under each type of street lamp. LPS lamps emit light over a narrow region of the light spectrum to which human photoreceptors are sensitive, hence objects that reflect light mainly outside this region appear dim or are not seen at all. HPS, LED and MH lamps emit light over a greater proportion of the light spectrum to which humans are sensitive (Fig. 1), hence more objects are easily detected under these lighting technologies because they are better discriminated in colour and brightness. The % λ_0.5_ range is a useful comparative index of the ability of animals to detect light reflected from ecologically relevant objects in their environment, because it represents the proportion of the visually detectable region of the light spectrum illuminated by a light source.

**Fig. 2:**
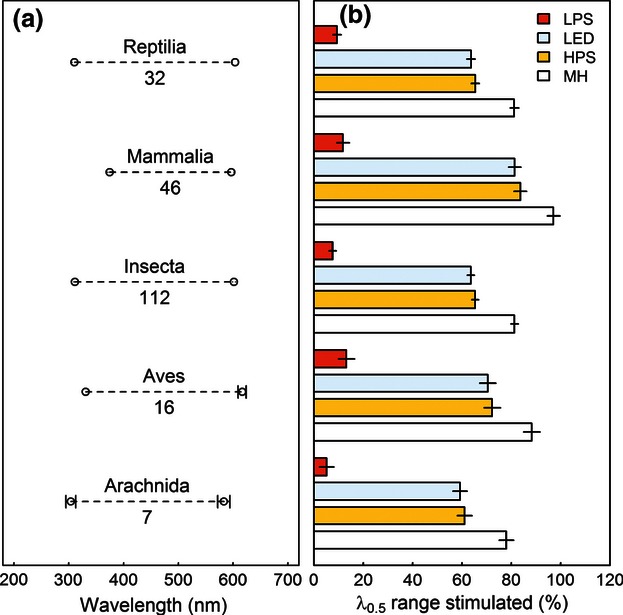
The percentage of the visual range stimulated by four contrasting street lighting technologies in five classes of animal. (a) The λ_0.5_ range of animals estimated for five classes. The average minimum and maximum wavelengths of half maximum visual pigment absorbance are denoted by points with error bars representing 95% credibility intervals estimated using MCMC regression. Values quoted under dashed lines represent the number of species on which derived values are based. (b) The percentage of the visual range at more than half maximum absorbance stimulated by each street light in each of five classes of animal. Means and 95% credibility intervals (error bars) were estimated using MCMC regression.

Values of % λ_0.5_ range were compared both between lighting technologies within each animal class, and between animal classes within each lighting technology using Markov Chain Monte Carlo regression (see below). To aid the interpretation of any patterns observed in the data, we also estimated the average maximum and minimum wavelengths at which the visual pigments of each animal group absorb more than half of the light entering the photoreceptor (max λ_0.5_ and min λ_0.5_) (Fig. 2a).

### Data collection (a) visual pigment λ_max_

Values of photoreceptor visual pigment λ_max_ were collected for 248 species of animal through an extensive literature search (see Table S1), and used to model the α and the β absorbance curves of the corresponding visual pigments using a standard formula (Govardovskii *et al*., 2000). Values of min λ_0.5_, max λ_0.5_ and λ_0.5_ range were then calculated for each of the 213 species. The remaining 35 species were omitted from the analysis due to missing values of λ_max_ for the visual pigments of known photoreceptors. For example, while the majority of insects possess UV photoreceptor cells, they have not been characterized in every studied species due to technological limitations (Bernard & Stavenga, 1979; Peitsch *et al*., 1992; Briscoe & Chittka, 2001). In species of insect for which the spectral sensitivity functions were available separately for females and males, either the sex for which fewer visual pigment λ_max_ wavelengths were quantified was omitted from the analysis, or if the number of visual pigments quantified was identical between sexes the male was omitted from the analysis. New World primates of the same species can be either dichromatic or trichromatic (Jacobs & Deegan, 2003). To prevent duplicating results for any one species, it was assumed that all individuals of each polymorphic species were trichromatic. The short, medium and long wave sensitive photoreceptors of birds and some diurnal reptiles are associated with oil droplets which alter the transmittance of light to the visual pigment and change the maximum wavelengths at which these pigments half maximally absorb light (UV sensitive photoreceptors and all photoreceptors in nocturnal reptiles possess clear oil droplets which do not affect the transmittance of light to the visual pigment; e.g., Hart & Vorobyev, 2005). For the birds and diurnal reptiles, changes in the absorbance curves of short, medium and long wavelength photoreceptors due to oil droplet transmittance were modelled prior to the estimation of min λ_0.5_ and max λ_0.5_ using the method outlined by Hart & Vorobyev (2005) and published values of oil droplet cut-off wavelengths (λ_cut_) or wavelengths at half maximum absorbance (λ_mid_) (see Table S1). In a few species, lens absorption produces a cut-off effect slightly limiting the visual range, but it has not been measured widely across species, therefore it was not included.

### Data collection (b) light spectra

The spectral compositions of four glass housed street lamps, one representative of each of the LPS (35W Thorn Beta 5 installed 12/2009), HPS (250W ZX3, Urbis installed 07/2008), LED (120W Ledway, Ruud installed 11/2010) and MH (45W Evolo lantern, Urbis installed 12/2009) technologies, were collected from municipal lighting installations in Cornwall, UK. While some variation exists in the exact spectra emitted by different makes and models of each technology, our selection was representative of the common differences between these technologies (narrow vs. broad spectrum, and UV vs. non-UV emissive). Light spectra were quantified using a MAYA2000-Pro spectrometer collecting light from a CC-3-UV-S cosine corrector connected via a 1000 μm fibre optic cable (Ocean Optics). The cosine corrector was held at ground level during measurements to capture the light spectra that most animals are likely to be exposed to. The resulting light spectra were used to quantify the region over which each lamp technology emits light (λ_light_ range).

### Data analysis

Photoreceptor signals are mainly determined by the maximum absorption of the photopigment at the wavelength λ_max_, and a photoreceptor's sensitivity decreases steeply with increasing distance from this peak sensitivity wavelength (Fig. 1). Visual systems have mostly evolved sets of receptors where sensitivities are well separated to avoid overlapping within the receptor's most sensitive range, usually between the half maximally sensitive (λ_0.5_) and λ_max_. Such spacing of photoreceptor sensitivities enables the effective coding of colours and increases an animal's ability to discriminate and recognize colours. Visual performance is influenced to a much lesser extent by the absorption of light in the low-sensitivity wavelength range. Therefore, the region of the light spectrum to which each species is more than half maximally sensitive (λ_0.5_ range) was determined as the visual range. The percentage of λ_0.5_ range stimulated by each street lamp technology (% λ_0.5_ range) was then estimated as the fraction of the λ_0.5_ range overlapped by the λ_light_ range (Fig. 1).

Means and 95% credibility interval values of min λ_0.5_, max λ_0.5_ and % λ_0.5_ range were estimated for each animal class perceiving light emitted from each type of street lamp using zero intercept Markov Chain Monte Carlo regression (MCMCregress; CRAN: MCMCpack; Martin *et al*., 2010) (1001 : 11000 iterations). Means and 95% credibility intervals of the difference in % λ_0.5_ range between street lamp types were estimated separately for each animal class, and separately for each street lamp type when comparing between animal classes, using MCMC regression with a fitted intercept (1001 : 11000 iterations). The resulting pairwise comparisons were interpreted in a manner analogous to parametric pairwise comparison tests. Where the credibility intervals of the difference between two street lamp types or animal classes did not bound 0, there is a 95% probability that they are different.

## Results

The results indicate that the four street lighting technologies can be divided into three categories based on how likely they are to facilitate the detection of objects reflecting light in different regions of the spectrum (Table 1; Fig. 2b): narrow spectrum lamps which do not emit UV light (LPS), broad spectrum lamps which do not emit UV light (HPS and LED) and broad spectrum lamps which do emit UV light (MH). There was a greater than 95% probability that the narrow spectral range of light emitted by LPS lamps stimulated the smallest proportion of the light spectrum to which animals are sensitive (Table 1) spanning from 5 ± 3.66% λ_0.5_ range in the arachnids to 13.1 ± 2.4% λ_0.5_ range in the birds (Fig. 2b). Metal Halide (MH) lamps stimulated the largest proportion of the light spectrum to which animals are sensitive spanning from 77.9 ± 5.4% λ_0.5_ range in the arachnids to 97.1 ± 2.1% λ_0.5_ range in the mammals (Fig. 2b). There was a greater than 95% probability that MH lamps stimulated a larger percentage of the λ_0.5_ range than each of the remaining lighting technologies (Table 1). HPS and LED lighting technologies stimulate similar percentages of the λ_0.5_ range in all classes of animal studied (Table 1). The broad emission spectra of these technologies stimulate a higher percentage of the λ_0.5_ range in comparison to LPS lamps with greater than 95% probability (Table 1) in all five animal classes, but to a lesser extent than MH lamps (Table 1; Fig. 2b).

**Table 1:** The difference in the percentage of the visual range at greater than half maximum absorbance (% λ_0.5_ range) stimulated by each of the four contrasting street lighting technologies compared within five classes of animal

	Street lamp type			
	
Class		LPS	HPS	LED
Arachnida	HPS	55.9_(51.7,60.1)_		
	LED	54.1_(50.0,58.3)_	−1.8_(-6.0,2.3)_	
	MH	72.9_(68.6,77.0)_	16.9_(12.7,21.1)_	18.8_(14.5,22.9)_
Aves	HPS	59.2_(54.4,63.8)_		
	LED	57.4_(52.7,62.1)_	−1.8_(−6.5,2.9)_	
	MH	75.1_(70.4,79.9)_	16.0_(11.2,20.6)_	17.8_(13.0,22.5)_
Insecta	HPS	57.8_(55.7,59.8)_		
	LED	56.1_(54.0,58.1)_	−1.7_(−3.8,0.3)_	
	MH	73.7_(71.6,75.7)_	15.9_(13.8,18.0)_	17.7_(15.6,19.7)_
Mammalia	HPS	71.9_(68.2,75.5)_		
	LED	69.7_(66.0,73.3)_	−2.3_(−5.9,1.3)_	
	MH	85.4_(81.7,89.0)_	13.5_(9.7,17.1)_	15.7_(12.0,19.4)_
Reptiles	HPS	56.1_(53.6,58.5)_		
	LED	54.4_(51.9,56.8)_	−1.7_(−4.2,0.7)_	
	MH	71.9_(69.4,74.3)_	15.8_(13.3,18.2)_	17.5_(15.0,20.0)_

Values represent the mean difference and 95% credibility intervals of the difference (values in parentheses) in % λ_0.5_ range stimulated by each lamp type. Values are derived from the pairwise comparison outputs from Markov Chain Monte Carlo simulations performed between factor levels going across the table subtracted from factor levels going down the table. Where values in parentheses do not bound zero there is a 95% probability that the two factor levels are different (underlined results).

In addition to changing the ability of animals to detect light reflected from objects in general, the contrasting lighting technologies also affected the comparative ability of different taxonomic groups to detect light reflected from objects. LPS lamps stimulate more of the λ_0.5_ range of birds and mammals compared to arachnids, insects and reptiles with greater than 95% probability (Fig. 2b; Table 2). HPS, LED and MH lights, however, increase the number and magnitude of differences in % λ_0.5_ range between animal classes with a greater than 95% probability (Table 2). These differences are greatest between the mammals and the remaining animal classes under LED and HPS lamp types (Table 2) because mammals detect light over a narrower region of the light spectrum (Fig. 2a). Similarly, the λ_0.5_ range of birds extends less into the shorter wavelengths compared to insects, arachnids and reptiles (Fig. 2a), hence there was a greater than 95% probability that a higher percentage of the light spectrum detected by birds is stimulated under HPS, LED and MH lamps (Table 2).

**Table 2:** The difference in the percentage of the visual range at greater than half maximum absorbance (% λ_0.5_ range) stimulated by each of four contrasting street lighting technologies compared between five classes of animal

	Class				
	
Street lamp type		Arachnida	Aves	Insecta	Mammalia
LPS	Aves	8.0_(3.6,12.3)_			
	Insecta	2.5_(−1.3,6.2)_	−5.5_(−8.1,−3.0)_		
	Mammalia	6.7_(2.8,10.6)_	−1.3_(−4.1,1.4)_	4.2_(2.5,5.9)_	
	Reptilia	4.3_(0.3,8.3)_	−3.8_(−6.7,−0.8)_	1.8_(−0.2,3.7)_	−2.4_(−4.6,−0.2)_
HPS	Aves	11.3_(3.4,18.9)_			
	Insecta	4.4_(−2.4,11.0)_	−6.9_(−11.4,−2.3)_		
	Mammalia	22.7_(15.7,29.7)_	11.4_(6.4,16.4)_	18.3_(15.3,21.4)_	
	Reptilia	4.4_(−2.8,11.7)_	−6.8_(−12.1,−1.6)_	0.1_(−3.4,3.6)_	−18.2_(−22.2,−14.2)_
LED	Aves	11.3_(3.4,18.9)_			
	Insecta	4.4_(−2.3,11.1)_	−6.9_(−11.4,−2.3)_		
	Mammalia	22.2_(15.2,29.2)_	10.9_(5.9,15.9)_	17.8_(14.8,20.8)_	
	Reptilia	4.5_(−2.7,11.8)_	−6.8_(−12.1, −1.5)_	0.1_(−3.4,3.6)_	−17.7_(−21.7,−13.7)_
MH	Aves	10.3_(3.8,16.6)_			
	Insecta	3.4_(−2.3,8.9)_	−6.9_(−10.7,−3.2)_		
	Mammalia	19.2_(13.4,25.0)_	8.9_(4.8,13.0)_	15.8_(13.4,18.4)_	
	Reptilia	3.3_(−2.7,9.3)_	−7.0_(−11.4,−2.6)_	−0.1_(−2.9,2.8)_	−15.9_(−19.2,−12.6)_

Values represent the mean difference and 95% credibility intervals of the difference (values in parentheses) in % λ_0.5_ range stimulated by each street lamp type. Values were derived from the pairwise comparison outputs from Markov Chain Monte Carlo simulations performed between factor levels going across the table subtracted from factor levels going down the table. Where values in parentheses do not bound zero there is a 95% probability that the two factor levels are different (underlined results).

## Discussion

Our results suggest that the installation of broader spectrum lighting technologies in artificially lit habitats is likely to improve the ability of animals to detect light reflected from objects in their environment at night, and has the potential to generate greater disparities in this ability between different classes of animal. These improvements in object detection under broad spectrum street lights are likely to affect the execution of visually guided behaviours in animals, altering their normal activity times and spatially extending or fragmenting habitats. All three broad spectrum lighting technologies provided significant improvements in the % λ_0.5_ range in comparison to narrow spectrum LPS lamps. MH lamps provided the greatest improvements in all five taxonomic classes. Hence, where these are in use, a greater variety of objects reflecting light in different regions of the light spectrum will appear brighter and more colourful to animals compared with alternative street lamp technologies. While LPS lamps illuminate objects reflecting light across the smallest region of the light spectrum, our results suggest that in areas illuminated by LPS lamps, birds and mammals are better able to detect objects that reflect light in this region compared to arachnids, insects and reptiles. The introduction of broader spectrum technologies, however, increases the number, and the magnitude of the differences between animal classes, in the proportion of the visually detectable light spectrum illuminated, with mammals and birds displaying the largest improvements. Most mammals possess dichromatic vision spanning a less extended range of the light spectrum in comparison to birds, reptiles, arachnids and insects (Fig. 2a; see Table S1) that typically can detect light at wavelengths below 400 nm (UV) (Tovée, 1995; Briscoe & Chittka, 2001; Hart & Hunt, 2007; Osorio & Vorobyev, 2008). Birds do possess UV sensitive photoreceptors, but their sensitivity extends less into the shorter wavelengths compared to insects, arachnids and reptiles (Fig. 2a). Broad spectrum lamp types therefore stimulate a larger percentage of the λ_0.5_ range in mammals and birds in general, compared with other classes of animal, improving their ability to perform visually guided behaviours with greater acuity and potentially upsetting the balance of interspecific interactions.

Our results provide an overview of how shifting artificial light spectra are likely to affect visually guided behaviours in broad taxonomic groups of animal. However, the λ_0.5_ range of individual species can be variable within each taxonomic group, and therefore caution should be exercised when applying the results of a group in general to any one specific species within that group. For example, the number of photoreceptor types in insect eyes is variable between different orders (Table S1) giving rise to variation in the proportion of λ_0.5_ range illuminated by each type of artificial light. In addition, the number of species for which λ_max_ values are available in the literature varies between taxonomic groups (Table S1), and while the main results of this study are unlikely to be affected, the λ_0.5_ range will inevitably adjust as data become available for more species and additional photoreceptors in those groups which are not currently well investigated (for example the arachnids). These results are not therefore conclusive, rather they should be considered as a platform of predictions which incentivises further studies into the impact of broadening artificial light spectra on visually guided behaviours in animals.

The ecological impacts of artificially lighting the nocturnal environment are increasingly being recognized (Frank, 2006; Stone *et al*., 2012; Titulaer *et al*., 2012), with some studies drawing attention to the potential impact of shifting spectral signatures (Eisenbeis, 2006; Stone *et al*., 2012). This study has highlighted that such changes may be affecting visually guided behaviours in species across the animal kingdom. The range of potential impacts are diverse and may include extending the times of foraging and sexual competition of diurnal and crepuscular animals into the night (Robertson & Monteiro, 2005; Somanathan *et al*., 2009; Titulaer *et al*., 2012), improving both prey detection and predator avoidance (Roth & Kelber, 2004), changing the ability of organisms to navigate around their environment (Warrant *et al*., 2004, Somanathan *et al*., 2008; Stone *et al*., 2009; van Langevelde *et al*., 2011) and affecting the ability of pollinating species to detect nectar resources (Kelber *et al*., 2002; Hempel de Ibarra & Vorobyev, 2009). Whether broadening artificial light spectra will elicit positive or negative species responses is likely to depend on the species and the behaviour being considered. For example, the presence of LED lighting increases feeding rates in nesting Great Tits *Parus major* (Titulaer *et al*., 2012), while the bat *Rhinolophus hipposideros* avoids areas lit by HPS and LED lighting (Stone *et al*., 2009, 2012) potentially due to perceived predation risk (Rydell, 1992). Metal Halide (MH) lamps are likely to provide the largest improvements in animal vision because they emit light that is both broad and contains UV in its spectral composition. Many of the above tasks depend on the perception of UV light reflected from objects by animals that can detect light at these wavelengths. Hence, the introduction of broader spectrum lighting technologies containing UV may have more profound consequences for biological systems than non-UV broad spectrum lighting technologies. All three broad spectrum technologies, however, create larger disparities in % λ_0.5_ between animal groups compared with narrow spectrum LPS lamps, and so have greater potential to alter the balance of interspecific interactions in the environment. Evaluating the direct environmental impacts of each of these different lamp types is clearly essential in a world where the artificially lit night-time environment is increasingly becoming ‘white’.

## Supporting Information

**Table S1.** The wavelengths of maximum absorbance (λ_max_) of the visual pigments contained within the photoreceptors of animal eyes.
