# Supplementary material for: Artificial light pollution: are shifting spectral signatures changing the balance of species interactions?
Source: Glob Chang Biol. 2013 Mar 12;19(5):1417–23. doi: 10.1111/gcb.12166 (PMC3657119; doi:10.1111/gcb.12166)
Supplement: Supplementary file 1 [file gcb0019-1417-SD1.pdf]

Table S1. The wavelengths of maximum absorbance (λ_{max}) of the visual pigments contained within the photoreceptors of animal eyes.

Phylum	Class	Order	Family	Species	λmax[1]	Source
Arthropoda	Arachnida	Araneae	Araneidae	*Argiope amoena*	360,490,540	(Yamashita & Tateda 1978)
				Argiope bruennichi	360,490,540	(Yamashita & Tateda 1978)
			Ctenidae	*Cupiennius salei*	340,480,520	(Walla *et al.* 1996)
			Lycosidae	*Lycosa sp.*	365,510	(DeVoe 1972)
			Salticidae	*Menemerus confusus*	360,490,530,538,580	(Yamashita & Tateda 1976)
				Phidippus regius	370,532	(De Voe 1975)
				Plexippus validus	360,520	(Blest *et al.* 1981)
	Insecta	Blattodea	Blattidae	*Periplaneta americana*	365,505	(Mote & Goldsmith 1970; Paul *et al.* 1986)
		Coleoptera	Carabidae	*Carabus auratus*	348,430,500,620	(Hasselmann 1962)
				Carabus nemoralis	348,430,500,620	(Hasselmann 1962)
			Coccinellidae	*Coccinella septempunctata*	370,520	(Lin & Wu 1992)
			Lampyridae	*Bicellonycha wickershamorum*	400,560	(Lall *et al.* 1988)
				Photinus collustrans	425,560	(Lall *et al.* 1988)
				Photinus macdermotti	400,575	(Lall *et al.* 1988)
				Photinus marginellus	375,565	(Lall *et al.* 1988)
				Photinus pyralis	375,570	(Lall *et al.* 1988)
				Photinus scintillans	450,580	(Lall *et al.* 1988)
				Photuris frontalis	350,560	(Lall *et al.* 1988)
				Photuris lucicrescens	350,440,550	(Lall *et al.* 1982)
				Photuris potomaca	375,560	(Lall *et al.* 1988)
		Diptera	Bibionidae	*Bibio marci*	520	(Burkhart & De LaMotte 1972)
			Calliphoridae	*Calliphora erythrocephala*	350,490	(Paul *et al.* 1986)
				Calliphora vicina	344,490	(Bernard & Stavenga 1979)
				Phaenicia sericata	480	(McCann & Arnett 1972)
			Chloropidae	*Chlorops sp.*	480	(Bernard & Stavenga 1979)
			Drosophilidae	*Drosophila melanogaster*	345,370,440,480,520	(Bernard & Stavenga 1979)
			Ephydridae	*Dimecoenia spinosa*	480	(Bernard & Stavenga 1979)
			Muscidae	*Musca domestica*	335,430,460,490,530	(Hardie 1986)
			Syrphidae	*Allograpta obliqua*	460	(Bernard & Stavenga 1979)
				Eristalis arbustorum	450	(Bernard & Stavenga 1979)

		Eristalis tenax	350,450,520	(Horridge *et al.* 1975)
		Syrphus sp.	440	(Bernard & Stavenga 1979)
		Toxomerus marginatus	450	(Bernard & Stavenga 1979)
	Tabanidae	*Haematopota sp.*	530	(Kirschfeld 1986)
Hemiptera	Notonectidae	*Notonecta glauca*	350,420,567	(Bruckmoser 1968)
		Notonecta irrorata	375,475,520	(Bennett & Ruck 1970)
		Notonecta isulata	375,475,520	(Bennett & Ruck 1970)
		Notonecta undulata	375,475,520	(Bennett & Ruck 1970)
Hymenoptera	Andrenidae	*Adrena florea*	340,412,536	(Peitsch *et al.* 1992)
		Callonychium petuniae	360,404,536,600	(Peitsch *et al.* 1992)
		Oxea flavescens	370,435,536	(Peitsch *et al.* 1992)
		Anthophora acervorum	348,428,528	(Peitsch *et al.* 1992)
	Apidae	*Apis mellifera* (female)	344,436,544	(Peitsch *et al.* 1992)
		Apis mellifera (male)	328,436,532	(Peitsch *et al.* 1992)
		Bombus affinis	525	(Bernard & Stavenga 1978)
		Bombus distinguendis	350,440,540	(Chittka *et al.* 2001)
		Bombus fervidus	350,450	(Bernard & Stavenga 1978)
		Bombus hortorum	353,436,544	(Meyer-Rochow 1980)
		Bombus hypnorum	524	(Peitsch *et al.* 1992)
		Bombus impatiens	352,450	(Bernard & Stavenga 1978)
		Bombus jonellus	336,432,544	(Peitsch *et al.* 1992)
		Bombus lapidarius	332,432,544	(Peitsch *et al.* 1992)
		Bombus monticola	336,440,544	(Peitsch *et al.* 1992)
		Bombus morio	352,428,548	(Peitsch *et al.* 1992)
		Bombus terrestris	328,428,536	(Peitsch *et al.* 1992)
		Lestrimelitta limao	536	(Peitsch *et al.* 1992)
		Melecta punctata	336,428,540	(Peitsch *et al.* 1992)
		Melipona marginata	340,450,540	(Peitsch *et al.* 1992)
		Melipona quadrifasciata	356,428,520	(Peitsch *et al.* 1992)
		Nomada alboguttata	428,512	(Peitsch *et al.* 1992)
		Partamona helleri	347,444,521	(Chittka *et al.* 1997)
		Schwarziana sp.	343,440,528	(Peitsch *et al.* 1992)
		Trigona spinipes	340,440,536	(Peitsch *et al.* 1992)
		Xylocopa brasilianorum	360,428,544	(Peitsch *et al.* 1992)
		Collectes fulgidus	340,532	(Peitsch *et al.* 1992)

	Colletidae	*Formica polyctena*	360,510	(Menzel & Knaut 1973)
	Crabronidae	*Philanthus triangulum*	344,444,524	(Peitsch *et al.* 1992)
		Cataglyphis bicolor	350,510	(Paul *et al.* 1986)
	Formicidae	*Anthidnium manicatum*	344,440,532	(Peitsch *et al.* 1992)
		<u>*Myrmecia gulosa*</u>	412,540	(Lieke 1981)
	Halictidae	<u>*Lasioglossum albipes*</u>	516	(Peitsch *et al.* 1992)
		<u>*Lasioglossum malachurum*</u>	442,528	(Peitsch *et al.* 1992)
	lchneumoidae	<u>*Ichneumon stramentarius*</u>	542	(Peitsch *et al.* 1992)
		<u>*Ichneumon spp.*</u>	524	(Peitsch *et al.* 1992)
	Megachilidae	*Chelostoma florisomne*	324,548	(Peitsch *et al.* 1992)
		Osmia rufa	344,432,560	(Peitsch *et al.* 1992)
	Siricidae	<u>*Urocerus gigas*</u>	524	(Peitsch *et al.* 1992)
	Sphecidae	<u>*Cerceris rybynensis* (female)</u>	436,516	(Peitsch *et al.* 1992)
		<u>*Cerceris rybynensis* (male)</u>	528	(Peitsch *et al.* 1992)
	Tenthredinidae	*Tenthredo campestris*	328,464,540	(Peitsch *et al.* 1992)
		<u>*Tenthredo scrophulariae*</u>	556,604	(Peitsch *et al.* 1992)
	Vespidae	<u>*Dorlichovespula norwegica*</u>	448,524	(Peitsch *et al.* 1992)
		Polistes dominulus	352,452,528	(Peitsch *et al.* 1992)
		Vespo crabro (female)	336,436,536	(Peitsch *et al.* 1992)
		<u>*Vespo crabro* (male)</u>	542	(Peitsch *et al.* 1992)
		Vespula germanica	336,432,544	(Peitsch *et al.* 1992)
		Vespula vulgaris	336,432,536	(Peitsch *et al.* 1992)
	Xiphydriidae	<u>*Xiphydria camelus*</u>	542	(Peitsch *et al.* 1992)
Lepidoptera	Epicopeiidae	*Epicopeia hainesii*	380,420,500	(Eguchi *et al.* 1982)
	Geometridae	*Arichanna gaschkevitchii*	360,500,540	(Eguchi *et al.* 1982)
	Hepialiidae	*Phassus excrescens*	380,460,520,580	(Eguchi *et al.* 1982)
	Hesperiidae	*Ochlodes venata*	380,460,520	(Eguchi *et al.* 1982)
		Parnarna guttata	380,460,520	(Eguchi *et al.* 1982)
	Lycaenidae	*Celastrina argiolus*	380,440,560	(Eguchi *et al.* 1982)
		Lycaena dorcas	360,437,500,568	(Bernard & Remington 1991)
		Lycaena heteronea	360,437,500,568	(Bernard & Remington 1991)
		Lycaena nivalis	361,438,501,575	(Bernard & Remington 1991)
		Lycaena phlaeas	400,540,600	(Eguchi *et al.* 1982)
		Lycaena rubidus	360,437,500,568	(Bernard & Remington 1991)
		Pseudozizeeria maha	400,520,580	(Eguchi *et al.* 1982)

Noctuidae	*Anadevidia peponis*	420,460,500	(Eguchi *et al.* 1982)
	Mamestra brassicae	360,460,540,580	(Ichikawa & Tateda 1982)
	Spodoptera exempta	355,465,515,560	(Langer *et al.* 1979)
Nymphalidae	*Aglais urticae*	380,460,530	(Steiner *et al.* 1987)
	Argynnis ruslana	380,440,560,620	(Eguchi *et al.* 1982)
	Fabriciana adippe	380,460,520,580	(Eguchi *et al.* 1982)
	Heliconius erato	370,470,570	(Weller & Pashley 1995)
	Heliconius numata	390,460,540	(Struwe 1972b, a)
	Heliconius sara	370,470,550	(Struwe 1972b)
	Minois dryas	380,460,520	(Eguchi *et al.* 1982)
	Neope goschkevitschii	380,460,520	(Eguchi *et al.* 1982)
	Nymphalis xanthomelas	380,460,500,560	(Eguchi *et al.* 1982)
	Pararge aegeria	360,460,530	(Paul *et al.* 1986)
	Polygonia calbum	380,520,560,620	(Eguchi *et al.* 1982)
	Vanessa cardui	360,470,530	(Bernard 1979; Briscoe *et al.* 2003)
Papilionidae	*Atrophaneura alcinous*	420,460,520,600	(Eguchi *et al.* 1982)
	Graphium sarpedon	380,460,560,600	(Eguchi *et al.* 1982)
	Papilio aegeus	360,450,540,610	(Matic 1983)
	Papilio bianor	420,460,520,580	(Eguchi *et al.* 1982)
	Papilio maackii	380,460,520,580	(Eguchi *et al.* 1982)
	Papilio machaon	380,460,520,580	(Eguchi *et al.* 1982)
	Papilio protenor	420,460,520	(Eguchi *et al.* 1982)
	Papilio xuthus	360,390,490,520,600	(Eguchi *et al.* 1982; Arikawa *et al.* 1987)
Pieridae	*Colias erate*	400,520,560	(Eguchi *et al.* 1982)
	Gonepteryx aspasia	380,460,560,600	(Eguchi *et al.* 1982)
	Pieris brassicae	360,455,560,620	(Paul *et al.* 1986; Steiner *et al.* 1987)
	Pieris melete	400,480,540,600	(Eguchi *et al.* 1982)
	Pieris rapae	340,450,540,600	(Bernard & Stavenga 1979; Ichikawa & Tateda 1982)
Pyralidae	*Amyelois transitella*	350,430,530	(Eguchi *et al.* 1982; Bernard *et al.* 1984)
	<u>*Galleria mellonella*</u>	510	(Goldman *et al.* 1975)
Saturniidae	*Actias artemis aliena*	380,460,540,580	(Eguchi *et al.* 1982)
	Antheraea polyphemus	360,450,525	(Langer *et al.* 1986)
	Saia cynthia ricini	380,400,480,520,540	(Eguchi *et al.* 1982)
Sphingidae	*Ampelophaga rubiginosa*	460,540,580	(Eguchi *et al.* 1982)

				Callambulyx tatarinovii	380,460,540,580	(Eguchi *et al.* 1982)
				Deilephila elpenor	345,440,520	(Hamdorf *et al.* 1971; Hoglund *et al.* 1973; Schwemer & Paulsen 1973; Schlecht 1979)
				Macroglossum stellatarum	348,430,500	(Hasselmann 1962)
				Manduca sexta	345,440,525	(White *et al.* 1983)
				Marumba sperchius	460,540,600	(Eguchi *et al.* 1982)
		Neuroptera	Ascalaphidae	*Libelloides macaronius*	350,530	(Gogala 1967; Paul *et al.* 1986)
		Odonata	Aeshnidae	*Aeschna cyanea*	356,420,519	(Autrum & Kolb 1968)
				Aeshna tuberculifera	358,501	(Chappell & DeVoe 1975)
				Anax junius	354,442,503	(Chappell & DeVoe 1975)
				Libellula pulchella	360,500	(Chappell & DeVoe 1975)
			Corduliidae	*Hemicordulia tau*	330,410,460,525,630	(Yang & Osorio 1991)
			Libellulidae	*Sympetrum rubicundulum*	340,410,490,540,620	(Meinertzhagen *et al.* 1983)
		Orthoptera	Acrididae	*Locusta migratoria*	360,430,530	(Vishnevskaya & Shura-Bura 1990)
			Blattidae	*Gryllus bimaculatus*	332,445,515	(Zufall *et al.* 1989)
				Gryllus campestris	340,439,520	(Zufall *et al.* 1989)
			Romaleidae	*Romalea microptera*	360,510	(Bruckler & Williams 1981)
Chordata	Aves[1]	Anseriformes	Anatidae	*Anas platyrhynchos*	415,452,506,567$_{(C,445,506,561)a}$	(Jane & Bowmaker 1988; Hart & Vorobyev 2005)
		Galliformes	Phasianidae	*Coturnix coturnix japonica*	418,450,505,567$_{(C,446,511,566)a}$	(Bowmaker *et al.* 1993; Hart & Vorobyev 2005)
				Gallus gallus	418,453,507,571$_{(C,443,505,561)a}$	(Bowmaker *et al.* 1997; Hart & Vorobyev 2005)
				Pavo cristatus	424,458,505,567$_{(C,449,511,569)a}$	(Hart 2002; Hart & Vorobyev 2005)
		Passeriformes	Estrildidae	*Amadina fasciata*	370,447,500,563$_{(C,423,516,575)a}$	(Hart *et al.* 2000a; Hart & Vorobyev 2005)
			Paridae	*Cyanistes caeruleus*	372,449,502,563$_{(C,413,508,573)a}$	(Hart *et al.* 2000b; Hart & Vorobyev 2005)
			Passeroidea	*Erythrura gouldiae*	370,440,500,562$_{(C,422,513,572)a}$	(Hart *et al.* 2000a; Hart & Vorobyev 2005)
				Lonchura maja	373,446,500,562$_{(C,422,510,567)a}$	(Hart *et al.* 2000a; Hart & Vorobyev 2005)
				Neochmia modesta	373,442,500,565$_{(C,415,514,568)a}$	(Hart *et al.* 2000a; Hart & Vorobyev 2005)
				Serinus canaria	363,440,501,567$_{(C,414,506,578)a}$	(Das *et al.* 1999; Hart & Vorobyev 2005)
			Sturnidae	*Sturnus vulgaris*	362,449,504,563$_{(C,399,515,573)a}$	(Hart *et al.* 1998; Hart & Vorobyev 2005)
			Timaliidae	*Leiothrix lutea*	355,454,499,568$_{(C,392,506,566)a}$	(Maier & Bowmaker 1993; Hart & Vorobyev 2005)
			Turdidae	*Turdus merula*	373,454,504,557$_{(C,414,515,570)a}$	(Hart *et al.* 2000b; Hart & Vorobyev 2005)

	Procellariiformes	Procellariidae	*Puffinus pacificus*	406,450,503,566$_{(C,445,506,562)a}$	(Hart 2004; Hart & Vorobyev 2005)
	Psittaciformes	Psittacidae	*Melopsittacus undulatus*	371,440,499,566$_{(C,411,507,566)a}$	(Bowmaker *et al.* 1997; Hart & Vorobyev 2005)
	Struthioniformes	Struthionidae	*Struthio camelus*	405,445,506,570$_{(C,417,506,552)a}$	(Wright & Bowmaker 2001; Hart & Vorobyev 2005)
Mammalia	Artiodactyla	Bovidae	*Bos taurus*	455,554	(Jacobs *et al.* 1998)
			Capra hircus	444,552	(Jacobs *et al.* 1998)
			Ovis aries	446,552	(Jacobs *et al.* 1998)
		Cervidae	*Dama dama*	453,542	(Jacobs *et al.* 1994)
			Odocoileus virginianus	457,537	(Jacobs *et al.* 1994)
		Suidae	*Sus scrofa*	439,556	(Neitz & Jacobs 1989)
	Carnivora	Canidae	*Canis lupus familiaris*	431,555	(Jacobs *et al.* 1993b)
			Urocyon littoralis	432,555	(Jacobs *et al.* 1993b)
			Vulpes vulpes	438,555	(Jacobs *et al.* 1993b)
		Felidae	*Felis catus*	450,555	(Crocker *et al.* 1980; Jacobs & Neitz 1986)
		Mustelidae	*Mustela putorius furo*	430,558	(Calderone & Jacobs 2003)
		Procyonidae	*Nasua nasua*	430,555	(Jacobs & Deegan 1992)
			Potos flavus	555	(Jacobs & Deegan 1992)
			Procyon lotor	560	(Jacobs & Deegan 1992)
	Didelphimorphia	Didelphidae	*Didelphis virginiana*	562	(Jacobs & Williams 2010)
	Lagomorpha	Leporidae	*Oryctolagus cuniculus*	425,523	(Nuboer *et al.* 1983)
	Perissodactyla	Equidae	*Equus caballus*	428,539	(Carroll *et al.* 2001)
	Primates	Aotidae	*Aotus trivirgatus*	543	(Jacobs *et al.* 1993a)
		Cebidae	*Callithrix jacchus jacchus* (trichromat)	423,548,564	(Travis *et al.* 1988; Tovée *et al.* 1992)
			Callithrix jacchus jacchus (dichromat)	423,558	(Travis *et al.* 1988; Tovée *et al.* 1992)
			Cebus spp. (trichromat)	426,537,563	(Jacobs & Deegan 2003)
			Cebus spp. (dichromat)	426,550	(Jacobs & Deegan 2003)
			Leontopithecus rosalia rosalia (trichromat)	430,548,561	(Jacobs & Deegan 2003)
			Leontopithecus rosalia rosalia (dichromat)	430,557	(Jacobs & Deegan 2003)
			Saguinus fuscicollis (trichromat)	440,540,590	(Jacobs *et al.* 1987)
			Saguinus fuscicollis (drichromat)	445,549	(Jacobs *et al.* 1987)
			Sagiunus midas (dichromat)	428,550	(Jacobs & Deegan 2003)
			Saguinus oedipus (trichromat)	428,546,555	(Jacobs & Deegan 2003)
			Saguinus oedipus (dichromat)	428,551	(Jacobs & Deegan 2003)

			Saimiri sciureus (trichromat)	434,550,558	(Jacobs & Neitz 1987)
			Saimiri sciureus (dichromat)	434,551	(Jacobs & Neitz 1987)
		Cercopithecidae	*Cercopithecus cephus*	432,533,565	(Bowmaker *et al.* 1991)
			Cercopithecus diana	432,531,566	(Bowmaker *et al.* 1991)
			Cercopithecus petaurista	424,534,563	(Bowmaker *et al.* 1991)
			Chlorocebus aethiops	434,535,566	(Bowmaker *et al.* 1991)
			Erythrocebus patas	432,533,566	(Bowmaker *et al.* 1991)
			Macaca fascicularis	430,531,561	(Baylor *et al.* 1984, 1987)
			Macaca mulatta	434,536,562	(Bowmaker *et al.* 1991)
			Miopithecus talapoin	429,533,564	(Bowmaker *et al.* 1991)
			Papio papio	431,536,564	(Bowmaker *et al.* 1991)
		Hominidae	*Homo sapiens*	419,531,558	(Dartnall *et al.* 1983)
		Lemuridae	*Eulemur fulvus fulvus*	437,545	(Jacobs & Deegan 1993)
			Lemur catta	437,545	(Jacobs & Deegan 1993)
		Pitheciidae	*Pithecia pithecia* (trichromat)	428,537,563	(Jacobs & Deegan 2003)
			Pithecia pithecia (dichromat)	428,565	(Jacobs & Deegan 2003)
	Proboscidea	Elephantidae	*Elephas maximus*	419,552	(Yokoyama *et al.* 2005)
			Loxodonta africana	419,552	(Yokoyama *et al.* 2005)
	Rodentia	Caviidae	*Cavia porcellus*	400,530	(Parry & Bowmaker 2002)
		Cricetidae	*Mesocricetus auratus*	508	(Calderone & Jacobs 1999; Williams & Jacobs 2008)
			Mesocricetus brandti	506	(Williams & Jacobs 2008)
			Phodopus sungorus	360,500	(Calderone & Jacobs 1999; Williams & Jacobs 2008)
		Geomyidae	*Thomomys bottae*	367,505	(Williams *et al.* 2005)
		Muridae	*Meriones unguiculatus*	360,493	(Jacobs *et al.* 1991; Jacobs & Deegan Ii 1994)
			Mus musculus	359,511	(Jacobs *et al.* 1991)
			Rattus norvegicus	359,511	(Jacobs *et al.* 1991)
		Sciuridae	*Sciurus carolinensis*	444,543	(Blakeslee *et al.* 1988)
			Spermophilus beecheyi	437,519	(Jacobs *et al.* 1985)
			Spermophilus lateralis	435,520	(Kraft 1988)
			Spermophilus tridecemlineatus	437,517	(Jacobs *et al.* 1985)
	Scandentia	Tupaiidae	*Tupaia glis*	445,555	(Bowmaker *et al.* 1991)
Reptilia[2]	Squamata	Agamidae	*Ctenophorus ornatus*	$440,493,571_{(470,470,470)a}$	(Barbour *et al.* 2002)
		Chamaeleonidae	*Chamaeleo dilepis*	$383,444,481,499,568,586,605_{(C,390,493,493,486,486,486)a}$	(Bowmaker *et al.* 2005)

	Furcifer pardalis	375,444,490,559,575,604$_{(C,390,493,493,486,486,487)a}$	(Bowmaker *et al.* 2005)
Colubridae	*Boa constrictor*	357,549$_{(C,C)}$	(Sillman *et al.* 2001)
	Masticophis flagellum	362,457,558$_{(C,C,C)}$	(Macedonia *et al.* 2009)
	Python regius	360,551$_{(C,C)}$	(Davies *et al.* 2009)
	Thamnophis sirtalis sirtalis	358,481,554$_{(C,C,C)}$	(Sillman *et al.* 1997)
	Xenopeltis unicolor	361,550$_{(C,C)}$	(Davies *et al.* 2009)
Gekkonidae	*Cyrtopodion caspium*	365,534,452$_{(C,C,C)}$	(Govardovskiî *et al.* 1984)
	Cyrtopodion fedtschenkoi	365,535,451$_{(C,C,C)}$	(Govardovskiî *et al.* 1984)
	Cyrtopodion kotschyi	365,537,460$_{(C,C,C)}$	(Govardovskiî *et al.* 1984)
	Gekko gecko	364,467,521$_{(C,C,C)}$	(Loew 1994)
	Hemidactylus frenatus	365,520,466$_{(C,C,C)}$	(Crescitelli *et al.* 1977)
	Hemidactylus garnotii	363,464,521$_{(C,C,C)}$	(Loew *et al.* 1996)
	Hemidactylus platyurus	365,527,465$_{(C,C,C)}$	(Crescitelli *et al.* 1977)
	Hemidactylus turcicus	366,467,526$_{(C,C,C)}$	(Loew *et al.* 1996)
	Ptychozoon lionotum	365,523,470$_{(C,C,C)}$	(Crescitelli *et al.* 1977)
	Teratoscincus scincus	365,452,533$_{(C,C,C)}$	(Loew *et al.* 1996)
Iguanidae	*Anolis bahorucoensis*	365,450,500,569$_{(C,397,450,500)a}$	(Loew *et al.* 2002)
	Anolis conspersus	365,460,500,562$_{(C,368,475,515)a}$	(Loew *et al.* 2002)
	Anolis cristatellus	365,458,492,562$_{(C,371,463,507)a}$	(Loew *et al.* 2002)
	Anolis evermanni	364,460,490,565$_{(C,380,500,515)a}$	(Loew *et al.* 2002)
	Anolis extremus	365,451,487,566$_{(C,393,442,488)a}$	(Loew *et al.* 2002)
	Anolis grahami	365,460,495,565$_{(C,382,451,505)a}$	(Loew *et al.* 2002)
	Anolis gundlachi	365,450,490,564$_{(C,370,450,510)a}$	(Loew *et al.* 2002)
	Anolis krugi	365,448,490,562$_{(C,370,480,500)a}$	(Loew *et al.* 2002)
	Anolis lineatopus	366,449,498,560$_{(C,367,451,486)a}$	(Loew *et al.* 2002)
	Anolis pulchellus	367,446,495,565$_{(C,390,475,505)a}$	(Loew *et al.* 2002)
	Anolis sagrei	365,460,495,567$_{(C,376,475,510)a}$	(Loew *et al.* 2002)
	Anolis stratulus	366,454,494,564$_{(C,388,467,495)a}$	(Loew *et al.* 2002)
	Crotaphytus dickersonae	359,459,481,558$_{(C,373,521,489)a}$	(Macedonia *et al.* 2009)
	Platysaurus broadleyi	364,451,492,570,569$_{(C,380,518,518,476)b}$	(Fleishman *et al.* 2011)

Species omitted from the analysis are underlined. [1] Values in parenthesis provide the wavelengths of λ_{cut} (a) or λ_{mid}(b) of the oil droplets contained within the photoreceptors of some reptiles and birds. These values were used to adjust the visual pigment absorbance curves for oil droplet transmittance prior to deriving the minimum and maximum wavelengths of half maximum absorbance(min $\lambda_{0.5}$ and max$\lambda_{0.5}$). 'C' denotes oil droplets which are clear and do not affect the transmittance of light to the visual pigment. Reference sources are provided below.

References

Arikawa K., Inokuma K. & Eguchi E. (1987). Pentachromatic visual system in a butterfly. *Naturwissenschaften*, 74, 297-298.

Autrum H. & Kolb G. (1968). Spektrale empfindlichkeit einzelner sehzellen der aeschniden. *Journal of Comparative Physiology A*, 60, 450-477.

Barbour H.R., Archer M.A., Hart N.S., Thomas N., Dunlop S.A., Beazley L.D. & Shand J. (2002). Retinal characteristics of the ornate dragon lizard, *Ctenophorus ornatus*. *The Journal of Comparative Neurology*, 450, 334-344.

Baylor D.A., Nunn B.J. & Schnapf J.L. (1984). The photocurrent, noise and spectral sensitivity of rods of the monkey *Macaca fascicularis*. *The Journal of Physiology*, 357, 575-607.

Baylor D.A., Nunn B.J. & Schnapf J.L. (1987). Spectral sensitivity of cones of the monkey *Macaca fascicularis*. *The Journal of Physiology*, 390, 145-160.

Bennett R.R. & Ruck P. (1970). Spectral sensitivities of dark- and light-adapted *Notonecta* compound eyes. *Journal of Insect Physiology*, 16, 83-88.

Bernard G.D. (1979). Red-absorbing visual pigment of butterflies. *Science*, 203, 1125-1127.

Bernard G.D., Owens E.D. & Hurley A.V. (1984). Intracellular optical physiology of the eye of the pyralid moth *Amyelois*. *Journal of Experimental Zoology*, 229, 173-187.

Bernard G.D. & Remington C.L. (1991). Color vision in Lycaena butterflies: spectral tuning of receptor arrays in relation to behavioral ecology. *Proceedings of the National Academy of Sciences*, 88, 2783-2787.

Bernard G.D. & Stavenga D.G. (1978). Spectral sensitivities of retinular cells measured in intact, living bumblebees by an optical method. *Naturwissenschaften*, 65, 442-443.

Bernard G.D. & Stavenga D.G. (1979). Spectral sensitivities of retinular cells measured in intact, living flies by an optical method. *Journal of Comparative Physiology A*, 134, 95-107.

Blakeslee B., Jacobs G.H. & Neitz J. (1988). Spectral mechanisms in the tree squirrel retina. *Journal of Comparative Physiology A*, 162, 773-780.

Blest A.D., Hardie R.C., McIntyre P. & Williams D.S. (1981). The spectral sensitivities of identified receptors and the function of retinal tiering in the principal eyes of a jumping spider. *Journal of Comparative Physiology A*, 145, 227-239.

Bowmaker J., Loew E. & Ott M. (2005). The cone photoreceptors and visual pigments of chameleons. *Journal of Comparative Physiology A*, 191, 925-932.

Bowmaker J.K., Astell S., Hunt D.M. & Mollon J.D. (1991). Photosensitive and photostable pigments in the retinae of Old World monkeys. *Journal of Experimental Biology*, 156, 1-19.

Bowmaker J.K., Heath L.A., Wilkie S.E. & Hunt D.M. (1997). Visual pigments and oil droplets from six classes of photoreceptor in the retinas of birds. *Vision Research*, 37, 2183-2194.

Bowmaker J.K., Kovach J.K., Whitmore A.V. & Loew E.R. (1993). Visual pigments and oil droplets in genetically manipulated and carotenoid deprived quail: A microspectrophotometric study. *Vision Research*, 33, 571-578.

Briscoe A.D., Bernard G.D., Szeto A.S., Nagy L.M. & White R.H. (2003). Not all butterfly eyes are created equal: Rhodopsin absorption spectra, molecular identification, and localization of ultraviolet-, blue-, and green-sensitive rhodopsin-encoding mRNAs in the retina of *Vanessa cardui*. *The Journal of Comparative Neurology*, 458, 334-349.

Bruckler R.M. & Williams T.P. (1981). Adaptation properties of the ERG in the grasshopper *Romalea microptera*. *European Biophysics Journal*, 7, 205-208.

Bruckmoser P. (1968). Die spektrale empfindlichkeit einzelner sehzellen des rückenschwimmers *Notonecta glauca* L. (Heteroptera). *Journal of Comparative Physiology A*, 59, 187-204.

Burkhart D. & De LaMotte I. (1972). Electrophysiological studies on the eyes of Diptera, Mecoptera and Hymenoptera. In: *Information Processing in the Visual System of Arthropods* (ed. Wehner R). Springer-Verlag Berlin, pp. 137-45.

Calderone J.B. & Jacobs G.H. (1999). Cone receptor variations and their functional consequences in two species of hamster. *Visual Neuroscience*, 16, 53-63.

Calderone J.B. & Jacobs G.H. (2003). Spectral properties and retinal distribution of ferret cones. *Visual Neuroscience*, 20, 11-17.

Carroll J., Murphy C.J., Neitz M., Ver Hoeve J.N. & Neitz J. (2001). Photopigment basis for dichromatic color vision in the horse. *Journal of Vision*, 1.

Chappell R.L. & DeVoe R.D. (1975). Action spectra and chromatic mechanisms of cells in the median ocelli of dragonflies. *The Journal of General Physiology*, 65, 399-419.

Chittka L., Schorn J., de Souza J.M., Ventura D.F. & Camargo J.M.F. (1997). The nest entrance of the Amazonian bees *Partamona pearsoni* - a case where insects design own flight targets. *Proceedings of the International Colloquia on Social Insects*, 3-4, 107-116.

Chittka L., Spaethe J., Schmitt A. & Hickelsberger A. (2001). Adaptation, constraint, and chance in the evolution of flower color and pollinator color vision. In: *Cognitive Ecology of Pollination* (eds. Chittka L & Thompson J). Cambridge University Press Cambridge.

Crescitelli F., Dartnall H.J.A. & Loew E.R. (1977). The gecko visual pigments: a microspectrophotometric study. *The Journal of Physiology*, 268, 559-573.

Crocker R.A., Ringo J., Wolbarsht M.L. & Wagner H.G. (1980). Cone contributions to cat retinal ganglion cell receptive fields. *The Journal of General Physiology*, 76, 763-785.

Dartnall H.J.A., Bowmaker J.K. & Mollon J.D. (1983). Human visual pigments: Microspectrophotometric results from the eyes of seven persons. *Proceedings of the Royal Society of London. Series B.* , 220, 115-130.

Das D., Wilkie S.E., Hunt D.M. & Bowmaker J.K. (1999). Visual pigments and oil droplets in the retina of a passerine bird, the canary *Serinus canaria*: microspectrophotometry and opsin sequences. *Vision Research*, 39, 2801-2815.

Davies W.L., Cowing J.A., Bowmaker J.K., Carvalho L.S., Gower D.J. & Hunt D.M. (2009). Shedding Light on Serpent Sight: The Visual Pigments of Henophidian Snakes. *The Journal of Neuroscience*, 29, 7519-7525.

De Voe R.D. (1975). Ultraviolet and green receptors in principal eyes of jumping spiders. *The Journal of General Physiology*, 66, 193-207.

De Voe R.D. (1972). Dual sensitivities of cells in wolf spider eyes at ultraviolet and visible wavelengths of light. *The Journal of General Physiology*, 59, 247-269.

Eguchi E., Watanabe K., Hariyama T. & Yamamoto K. (1982). A comparison of electrophysiologically determined spectral responses in 35 species of Lepidoptera. *Journal of Insect Physiology*, 28, 675-682.

Fleishman L.J., Loew E.R. & Whiting M.J. (2011). High sensitivity to short wavelengths in a lizard and implications for understanding the evolution of visual systems in lizards. *Proceedings of the Royal Society B*.

Gogala M. (1967). Die spektrale Empfindlichkeit der Doppelaugen von *Ascalaphus macaronius* Scop. (Neuroptera, Ascalaphidae). *Journal of Comparative Physiology A*, 57, 232-243.

Goldman L.J., Barnes S.N. & Goldsmith T.H. (1975). Microspectrophotometry of rhodopsin and metarhodopsin in the moth *Galleria*. *The Journal of General Physiology*, 66, 383-404.

Govardovskiî V.I., Zueva L.V. & Lychakov D.V. (1984). Microspectrophotometric study of visual pigments in five species of geckos. *Vision Research*, 24, 1421-1423.

Hamdorf K., Hoglund G. & Langer H. (1971). Mikrophotometrische untersuchungen an der retinula des nachtschmetterlings *Deilphila elpenor*. *Verhandlungen der Deutschen Zoologischen Gesellschaft*, 65, 276-80.

Hardie R.C. (1986). The photoreceptor array of the dipteran retina. *Trends in Neurosciences*, 9, 419-423.

Hart N., Partridge J. & Cuthill I. (1998). Visual pigments, oil droplets and cone photoreceptor distribution in the european starling (*Sturnus vulgaris*). *Journal of Experimental Biology*, 201, 1433-46.

Hart N.S. (2002). Vision in the peafowl (Aves: *Pavo cristatus*). *Journal of Experimental Biology*, 205, 3925-3935.

Hart N.S. (2004). Microspectrophotometry of visual pigments and oil droplets in a marine bird, the wedge-tailed shearwater *Puffinus pacificus*: topographic variations in photoreceptor spectral characteristics. *Journal of Experimental Biology*, 207, 1229-1240.

Hart N.S., Partridge J.C., Bennett A.T.D. & Cuthill I.C. (2000a). Visual pigments, cone oil droplets and ocular media in four species of estrildid finch. *Journal of Comparative Physiology A*, 186, 681-694.

Hart N.S., Partridge J.C., Cuthill I.C. & Bennett A.T.D. (2000b). Visual pigments, oil droplets, ocular media and cone photoreceptor distribution in two species of passerine bird: the blue tit (*Parus caeruleus* L.) and the blackbird (*Turdus merula* L.). *Journal of Comparative Physiology A*, 186, 375-387.

Hart N.S. & Vorobyev M. (2005). Modelling oil droplet absorption spectra and spectral sensitivities of bird cone photoreceptors. *Journal of Comparative Physiology A*, 191, 381-392.

Hasselmann E.-M. (1962). Uber die relative spektrale empfindlichkeit von kafer-und schmetterlingsaugen bei verschiedenen helligkeiten. *Zoologische Jahrbuecher Physiology*, 69, 537-76.

Hoglund G., Hamdorf K., Langer H., Paulsen R. & Schwemer J. (1973). The photopigments in an insect retina. In: *Biochemistry and Physiology of Visual Pigments* (ed. Langer H). Springer-Verlag Berlin/Heidelberg/New York.

Horridge G.A., Mimura K. & Tsukahara Y. (1975). Fly photoreceptors II. Spectral and polarized light sensitivity in the drone fly *Eristalis*. *Proceedings of the Royal Society of London B. Biological Sciences*, 190, 225-237.

Ichikawa T. & Tateda H. (1982). Distribution of color receptors in the larval eyes of four species of lepidoptera. *Journal of Comparative Physiology A*, 149, 317-324.

Jacobs G. & Williams G. (2010). Cone pigments in a North American marsupial, the opossum (*Didelphis virginiana*). *Journal of Comparative Physiology A*, 196, 379-384.

Jacobs G.H. & Deegan Ii J.F. (1994). Sensitivity to ultraviolet light in the gerbil (*Meriones unguiculatus*): Characteristics and mechanisms. *Vision Research*, 34, 1433-1441.

Jacobs G.H., Deegan Ii J.F., Neitz J., Crognale M.A. & Neitz M. (1993a). Photopigments and color vision in the nocturnal monkey,*Aotus*. *Vision Research*, 33, 1773-1783.

Jacobs G.H. & Deegan J.F. (1992). Cone photopigments in nocturnal and diurnal procyonids. *Journal of Comparative Physiology A*, 171, 351-358.

Jacobs G.H. & Deegan J.F. (1993). Photopigments underlying color vision in ringtail lemurs (*Lemur catta*) and brown lemurs (*Eulemur fulvus*). *American Journal of Primatology*, 30, 243-256.

Jacobs G.H. & Deegan J.F. (2003). Cone pigment variations in four genera of new world monkeys. *Vision Research*, 43, 227-236.

Jacobs G.H., Deegan J.F., Crognale M.A. & Fenwick J.A. (1993b). Photopigments of dogs and foxes and their implications for canid vision. *Visual Neuroscience*, 10, 173-180.

Jacobs G.H., Deegan J.F. & Neitz J. (1998). Photopigment basis for dichromatic color vision in cows, goats, and sheep. *Visual Neuroscience*, 15, 581-584.

Jacobs G.H., Deegan J.F., Neitz J., Murphy B.P., Miller K.V. & Marchinton R.L. (1994). Electrophysiological measurements of spectral mechanisms in the retinas of two cervids: white-tailed deer (*Odocoileus virginianus*) and fallow deer (*Dama dama*). *Journal of Comparative Physiology A*, 174, 551-557.

Jacobs G.H. & Neitz J. (1986). Spectral sensitivity of cat cones to rapid flicker. *Experimental Brain Research*, 62, 446-448.

Jacobs G.H. & Neitz J. (1987). Inheritance of color vision in a New World monkey (*Saimiri sciureus*). *Proceedings of the National Academy of Sciences*, 84, 2545-2549.

Jacobs G.H., Neitz J. & Crognale M. (1985). Spectral sensitivity of ground squirrel cones measured with ERG flicker photometry. *Journal of Comparative Physiology A*, 156, 503-509.

Jacobs G.H., Neitz J. & Crognale M. (1987). Color vision polymorphism and its photopigment basis in a callitrichid monkey (*Saguinus fuscicollis*). *Vision Research*, 27, 2089-2100.

Jacobs G.H., Neitz J. & Deegan J.F. (1991). Retinal receptors in rodents maximally sensitive to ultraviolet light. *Nature*, 353, 655-656.

Jane S.D. & Bowmaker J.K. (1988). Tetrachromatic colour vision in the duck (*Anas platyrhynchos* L.):microspectrophotometry of visual pigments and oil droplets. *Journal of Comparative Physiology A*, 162, 225-235.

Kirschfeld K. (1986). Activation of visual pigment chromophore structure and function. In: *Life Sciences Research Report, 34: The Molecular Mechanism of Photoreception; Dahlem Workshop* (ed. Stieve H). Springer-Verlg Berlin, pp. 31-50.

Kraft T.W. (1988). Photocurrents of cone photoreceptors of the golden-mantled ground squirrel. *The Journal of Physiology*, 404, 199-213.

Lall A.B., Lord E.T. & Trouth C.O. (1982). Vision in the firefly *Photuris lucicrescens* (Coleoptera: Lampyridae): Spectral sensitivity and selective adaptation in the compound eye. *Journal of Comparative Physiology A*, 147, 195-200.

Lall A.B., Strother G.K., Cronin T.W. & Seliger H.H. (1988). Modification of spectral sensitivities by screening pigments in the compound eyes of twilight-active fireflies (Coleoptera: Lampyridae). *Journal of Comparative Physiology A*, 162, 23-33.

Langer H., Hamann B. & Meinecke C.C. (1979). Tetrachromatic visual system in the moth *Spodoptera exempta* (Insecta: Noctuidae). *Journal of Comparative Physiology A*, 129, 235-239.

Langer H., Schmeinck G. & Anton-Erxleben F. (1986). Identification and localization of visual pigments in the retina of the moth, *Antheraea polyphemus* (Insecta, Saturniidae). *Cell and Tissue Research*, 245, 81-89.

Lieke E. (1981). Graded and discrete receptor potentials in the compound eye of the Australian Bulldog-ant (*Myrmecia gulosa*). *Biological Cybernetics*, 40, 151-156.

Lin J.T. & Wu C.Y. (1992). A comparative study on the color vision of 4 coleopteran insects. *Bulletin of the Institute of Zoology Academia Sinica*, 31, 81-88.

Loew E.R. (1994). A third, ultraviolet-sensitive, visual pigment in the Tokay gecko (*Gekko gekko*). *Vision Research*, 34, 1427-1431.

Loew E.R., Fleishman L.J., Foster R.G. & Provencio I. (2002). Visual pigments and oil droplets in diurnal lizards. *Journal of Experimental Biology*, 205, 927-938.

Loew E.R., Govardovskii V.I., Rohlich P. & Szel A. (1996). Microspectrophotometric and immunocytochemical identification of ultraviolet photoreceptors in geckos. *Visual Neuroscience*, 13, 247-256.

Macedonia J.M., Lappin A.K., Loew E.R., McGuire J.A., Hamilton P.S., Plasman M., Brandt Y., Lemos-Espinal J.A. & Kemp D.J. (2009). Conspicuousness of Dickerson's collared lizard (*Crotaphytus dickersonae*) through the eyes of conspecifics and predators. *Biological Journal of the Linnean Society*, 97, 749-765.

Maier E.J. & Bowmaker J.K. (1993). Colour vision in the passeriform bird, *Leiothrix lutea* correlation of visual pigment absorbance and oil droplet transmission with spectral sensitivity. *Journal of Comparative Physiology A*, 172, 295-301.

Matic T. (1983). Electrical inhibition in the retina of the butterfly *Papilio*. I. Four spectral types of photoreceptors. *Journal of Comparative Physiology*, 152, 169-82.

McCann G.D. & Arnett D.W. (1972). Spectral and polarization sensitivity of the dipteran visual system. *The Journal of General Physiology*, 59, 534-558.

Meinertzhagen I.A., Menzel R. & Kahle G. (1983). The identification of spectral receptor types in the retina and lamina of the dragonfly *Sympetrum rubicundulum*. *Journal of Comparative Physiology A*, 151, 295-310.

Menzel R. & Knaut R. (1973). Pigment movement during light and chromatic adaptation in the retinula cells of *Formica polyctena* (Hymenoptera, Formicidae). *Journal of Comparative Physiology A*, 86, 125-138.

Meyer-Rochow V.B. (1980). Electrophysiologically determined spectral efficiencies of the compound eye and median ocellus in the bumblebee *Bombus hortorum tarhakimalainen* (hymenoptera, insecta). *Journal of Comparative Physiology A*, 139, 261-266.

Mote M.I. & Goldsmith T.H. (1970). Spectral sensitivities of color receptors in the compound eye of the cockroach *Periplaneta*. *Journal of Experimental Zoology*, 173, 137-145.

Neitz J. & Jacobs G.H. (1989). Spectral sensitivity of cones in an ungulate. *Visual Neuroscience*, 2, 97-100.

Nuboer J.F.W., Nuys W.M. & Wortel J.F. (1983). Cone systems in the rabbit retina revealed by ERG-null-detection. *Journal of Comparative Physiology A*, 151, 347-352.

Parry J.W.L. & Bowmaker J.K. (2002). Visual pigment coexpression in guinea pig cones: A microspectrophotometric study. *Investigative Ophthalmology & Visual Science*, 43, 1662-1665.

Paul R., Steiner A. & Gemperlein R. (1986). Spectral sensitivity of *Calliphora erythrocephala* and other insect species studied with Fourier Interferometric Stimulation (FIS). *Journal of Comparative Physiology A*, 158, 669-680.

Peitsch D., Fietz A., Hertel H., Souza J., Ventura D.F. & Menzel R. (1992). The spectral input systems of hymenopteran insects and their receptor-based colour vision. *Journal of Comparative Physiology A*, 170, 23-40.

Schlecht P. (1979). Colour discrimination in dim light: An analysis of the photoreceptor arrangement in the moth *Deilephila*. *Journal of Comparative Physiology A*, 129, 257-267.

Schwemer J. & Paulsen R. (1973). Three visual pigments in *Deilephila elpenor* (Lepidoptera, Sphingidae). *Journal of Comparative Physiology A*, 86, 215-229.

Sillman A.J., Govardovskii V.I., Rohlich P., Southard J.A. & Loew E.R. (1997). The photoreceptors and visual pigments of the garter snake (*Thamnophis sirtalis*): a microspectrophotometric, scanning electron microscopic and immunocytochemical study. *Journal of Comparative Physiology A*, 181, 89-101.

Sillman A.J., Johnson J.L. & Loew E.R. (2001). Retinal photoreceptors and visual pigments in *Boa constrictor imperator*. *Journal of Experimental Zoology*, 290, 359-365.

Steiner A., Paul R. & Gemperlein R. (1987). Retinal receptor types *Aglais urticae* and *Pieris brassicae* (Lepidoptera), revealed by analysis of the electroretinogram obtained with Fourier Interferometric Stimulation (FIS). *Journal of Comparative Physiology A*, 160, 247-258.

Struwe G. (1972a). Spectral sensitivity of single photoreceptors in the compound eye of a tropical butterfly (*Heliconius numata*). *Journal of Comparative Physiology A*, 79, 197-201.

Struwe G. (1972b). Spectral sensitivity of the compound eye in butterflies (*Heliconius*). *Journal of Comparative Physiology A*, 79, 191-196.

Tovée M.J., Bowmaker J.K. & Mollon J.D. (1992). The relationship between cone pigments and behavioural sensitivity in a new world monkey (*Callithrix jacchus jacchus*). *Vision Research*, 32, 867-878.

Travis D.S., Bowmaker J.K. & Mollon J.D. (1988). Polymorphism of visual pigments in a callitrichid monkey. *Vision Research*, 28, 481-490.

Vishnevskaya T.M. & Shura-Bura T.M. (1990). Spectral sensitivity of photoreceptors and spectral inputs to the neurons of the first optic ganglion in the locust (*Locusta migratoria*). In: *Sensory Systems and Communication in Arthropods* (eds. Gribakin FG, Wiese K & Popov AV). Birkhauser Verlag, pp. 106-11.

Walla P., Barth F.G. & Eguchi E. (1996). Spectral sensitivity of single photoreceptor cells in the eyes of the ctenid spider *Cupiennius salei* Keys. *Zoological Science*, 13, 199-202.

Weller S.J. & Pashley D.P. (1995). In Search of Butterfly Origins. *Molecular Phylogenetics and Evolution*, 4, 235-246.

White R.H., Brown P.K., Hurley A.K. & Bennett R.R. (1983). Rhodopsins, retinula cell ultrastructure, and receptor potentials in the developing pupal eye of the moth *Manduca sexta*. *Journal of Comparative Physiology A*, 150, 153-163.

Williams G. & Jacobs G. (2008). Absence of functional short-wavelength sensitive cone pigments in hamsters (*Mesocricetus*). *Journal of Comparative Physiology A*, 194, 429-439.

Williams G.A., Calderone J.B. & Jacobs G.H. (2005). Photoreceptors and photopigments in a subterranean rodent, the pocket gopher (*Thomomys bottae*). *Journal of Comparative Physiology A*, 191, 125-134.

Wright M.W. & Bowmaker J.K. (2001). Retinal photoreceptors of paleognathous birds: the ostrich (*Struthio camelus*) and rhea (*Rhea americana*). *Vision Research*, 41, 1-12.

Yamashita S. & Tateda H. (1976). Spectral sensitivities of jumping spider eyes. *Journal of Comparative Physiology A*, 105, 29-41.

Yamashita S. & Tateda H. (1978). Spectral sensitivities of the anterior median eyes of the orb web spiders, *Argiope bruennichii* and *A. amoena*. *Journal of Experimental Biology*, 74, 47-57.

Yang E.-C. & Osorio D. (1991). Spectral sensitivities of photoreceptors and lamina monopolar cells in the dragonfly, *Hemicordulia tau*. *Journal of Comparative Physiology A*, 169, 663-669.

Yokoyama S., Takenaka N., Agnew D.W. & Shoshani J. (2005). Elephants and human color-blind Deuteranopes have identical sets of visual pigments. *Genetics*, 170, 335-344.

Zufall F., Schmitt M. & Menzel R. (1989). Spectral and polarized light sensitivity of photoreceptors in the compound eye of the cricket (*Gryllus bimaculatus*). *Journal of Comparative Physiology A*, 164, 597-608.
